# Post-Traumatic Stress Symptoms among Lithuanian Parents Raising Children with Cancer

**DOI:** 10.3390/children7090116

**Published:** 2020-08-31

**Authors:** Irina Banienė, Nida Žemaitienė

**Affiliations:** Department of Health Psychology, Faculty of Public Health, Lithuanian University of Health Sciences, LT-47181 Kaunas, Lithuanian; nida.zemaitiene@lsmuni.lt

**Keywords:** post-traumatic stress symptoms, PTSD, child with cancer, parents

## Abstract

Background and objectives: The study aims to evaluate post-traumatic stress symptom expression among Lithuanian parents raising children with cancer, including social, demographic, and medical factors, and to determine their significance for the risk of developing post-traumatic stress disorder. Materials and methods: The study was carried out in two major Lithuanian hospitals treating children with oncologic diseases. The cross-sectional study included 195 parents, out of which 151 were mothers (77.4%) and 44 were fathers (22.6%). Post-traumatic stress symptoms were assessed using the Impact of Event Scale-Revised. To collect the sociodemographic, childhood cancer, and treatment data, we developed a questionnaire that was completed by the parents. Main study results were obtained using multiple linear regression. Results: A total of 75.4% of parents caring for children with cancer had pronounced symptoms of post-traumatic stress disorder. The female gender (*β* = 0.83, *p* < 0.001) was associated with an increased manifestation of symptoms, whilst higher parental education (*β* = −0.21, *p* = 0.034) and the absence of relapse (*β* = −0.48, *p* < 0.001) of the child’s disease reduced post-traumatic stress symptom expression. Conclusions: Obtained results confirmed that experiencing a child’s cancer diagnosis and treatment is extremely stressful for many parents. This event may lead to impaired mental health and increased post-traumatic stress disorder (PTSD) risk; hence, it is necessary to provide better support and assistance to parents of children with cancer.

## 1. Introduction

Various difficulties and challenges are a universal and inevitable part of human life. Nonetheless, some events can be particularly shocking, and have a strong impact on a person’s mental health, functioning, and adaptation to the environment. One of them is a child’s oncological disease [1].

Each year, approximately 300,000 children are globally diagnosed with cancer [2]. Even though the survival rate of childhood cancer improved in recent years, learning about the diagnosis of a child’s oncologic disease is often seen as the most shocking, stressful event that can affect the whole family [3]. When a child develops cancer, parents’ traumatic experience does not end with the diagnosis of the disease. Usually, a long and complex treatment that lasts one–three years on average awaits. In addition, regular follow-up appointments are often needed for several years after initial treatment to prevent the recurrence of cancer [4]. According to Muscara et al. [5], one of the main factors causing parents’ traumatic experience is the application of sometimes unpleasant and painful cancer treatment methods to the affected child. Parents of sick children feel fear, uncertainty about the future, helplessness, and anger at the perceived injustice, noticing a likely or imminent death of a loved child [6]. Stress due to the risk to the child’s life becomes the most significant cause of acute and prolonged anxiety, depression [6], and post-traumatic stress disorder (PTSD) symptoms [7]. This was further confirmed by other authors arguing that the diagnosis of a childhood cancer is considered as an event that can lead to parental PTSD [5,8,9,10].

The World Health Organization’s International Statistical Classification of Diseases and Related Health Problems-Tenth Edition (ICD-10) [11] defines PTSD (F43.1) as a delayed response to a stressful, unusually threatening, or catastrophic incident or situation (regardless of its duration) that is likely to cause extensive distress in nearly anyone. People who have PTSD may experience intense feelings of danger, fear, and helplessness, with subsequent symptoms of intrusion, avoidance, and hyperarousal [12]. Post-traumatic stress symptoms (PTSS) in parents of children with cancer are expressed in several ways. Parents often experience a child’s diagnosis and disease in an especially sensitive and painful manner, tend to avoid thoughts related to cancer, suppress emotions, and hide feelings from others. Moreover, states of hypersensitivity, anger, and/or persistent irritability are also common. They might struggle to cope with insomnia, obsessive thoughts, impaired cognitive function, and other psychological or emotional difficulties [13,14].

It is estimated that from 25% [5,14,15,16] to 89% [13] of parents caring for a child with oncologic disease experience the above-mentioned symptoms. These differences may arise due to divergence in study size and design, cultural differences in healthcare systems, and other factors. Most studies that examine the risk of developing PTSD symptoms among parents raising a child with cancer assessed sociodemographic factors (parental gender, age, education, etc.), and aspects related to the child’s disease and treatment (diagnosis, interval of time after diagnosis, treatment methods, etc.). Despite numerous studies in the field, many questions about the impact of childhood cancer on parents’ mental health remain to be answered. The significance of parental gender and education level on the severity of PTSD symptoms is still controversial. Some studies report no statistical significance of PTSD symptom expression between genders [14,17,18], while others argue that mothers tend to develop parental PTSD more often than fathers [19,20,21,22]. Concerning education level, research results diverge, demonstrating that both parents lacking education [14] and highly educated ones [19] experience stronger symptoms than the respective group does.

As this problem has not been studied in Lithuania, it is unknown what proportion of parents raising a child with cancer express PTSD symptoms and how sociodemographic and medical factors are contributing towards it. The obtained data will highlight the possible significance of the socio-cultural context for the risk of mental illness in parents of children with oncologic disease. It will also help to better anticipate and plan preventive measures for parents caring for children with cancer.

## 2. Materials and Methods

Children with cancer are treated in two Lithuanian hospitals: Vilnius University Hospital Santaros Clinics Children’s Hospital at the Center for Pediatric Oncology and Hematology, and Lithuanian University of Health Sciences Kaunas Clinics at the Pediatric Oncology and Hematology Division. Therefore, the study was carried out in these major Lithuanian hospitals. A cross-sectional survey design was used to achieve the research aims. In agreement with associated hospitals, the leading researcher met parents whose children were receiving treatment during the study period. The researcher presented the purpose of the study and invited the parents who met the inclusion criteria to participate. The respondents who signed the informed consent then privately filled out the study questionnaire and returned it in a sealed envelope to a designated area. The included study participants were literate Lithuanian citizens who had an under-18-years-old child diagnosed with cancer longer than one month ago. The exclusion criterion was the refusal to give informed consent. This research was approved by the Biomedical Research Committee (no. BE-2-44).

### 2.1. Research Tools

PTSS were assessed using the Lithuanian version [23] of the Impact of Event Scale-Revised (IES-R) [24]. The IES-R methodology was designed to assess the severity of three types of PTSS, avoidance, invasion, and hyperarousal that meet the criteria for PTSD diagnosis according to ICD-10-AM, and to determine the overall severity of PTSS.

The IES-R scale consists of 22 statements. When completing the scale, the study participants were asked to indicate the intensity of encountered difficulties over the past seven days regarding their child’s cancer diagnosis on a 5-point scale (0—no manifestation; 4—severe symptoms). Total IES-R score is an arithmetic mean of all statement values, whereas subscale IES-R score is a mean of statement values included in that scale.

A mean of ≥1.5 or overall score of 33 on the IES-R scale is a cut-off value indicating a possible PTSD diagnosis [25,26]. This criterion was implemented to this study to assess the general expression of PTSD symptoms and evaluate them as separate facets such as avoidance, invasion, and hyperarousal. In this study, the reliability and validity of internal IES-R consistency was high in total (Cronbach’s *α* = 0.92) and subscale scores (Avoidance = 0.80, Invasion = 0.89, Hyperarousal = 0.86). The methodology authors indicated that Cronbach’s *α* coefficients vary from 0.84 to 0.92 [24].

The questionnaire was designed by the study researchers. The questions on sociodemographic and medical data were based on a synthesis of analyzed scientific literature [14,17,18,19,21]. Parents were asked about their gender, age, education, place of residence, employment, subjectively assessed family financial situation, marital status, number of children in the family, age and gender of the child, cancer type and stage, disease duration, treatment method, and relapse. Some questions for quantitative data were open (e.g., parent age, number of children in the family, age of child with cancer); others were presented as multiple choice (e.g., education, marital status). Two additional questions addressing subjectively perceived treatment prognosis and parental involvement in childcare were formulated by the researchers as scaled questions. Parents were asked to indicate how they would rate the information about their child’s disease prognosis on a scale of 1 to 5, where 1—the prognosis provides little hope of stopping cancer, and 5—the prognosis provides much hope of stopping cancer. Respondents who marked 1–3 were categorized as perceiving the diagnosis as bad, and those who marked 4 and 5 as perceiving the diagnosis as good. To evaluate parental involvement in childcare, the participants were asked to mark how much time they spend for the care of the child with cancer on a scale from 1 to 5, where 1—daily, the whole day, 2—daily, a few hours, 3—a few times per week, 4—a few times per month, and 5—very rarely or never. Daily caretakers (questions 1, 2) were allocated to the direct involvement group and less frequently (questions 3–5) implicated parents were classified as involved indirectly.

### 2.2. Data Analysis

Quantitative variables that met the assumption of normality were evaluated by calculating means (M) and standard deviations (SD). If data were non-normally distributed, median (Md), minimal, and maximal values were assessed. The normality of distribution was tested by Kolmogorov–Smirnov compatibility criteria when the number of cases was greater than 50, and the Shapiro–Wilk test if sample size was smaller. Qualitative (categorical) variables were described by frequency and percentage.

Parametric criteria (Student’s *t*-test, ANOVA) were used for normally distributed variables, and nonparametric criteria for non-normally distributed variables (Mann–Whitney U test, Kruskal–Wallis test). The Student’s *t*-test and Mann–Whitney U test compared two independent samples (means or medians). ANOVA and the Kruskal–Wallis test were used for more than two independent samples (means or medians). The linear regression analysis of one variable was performed to determine whether social, demographic, and medical factors were statistically significant in predicting the severity of PTSD symptoms. The final linear regression model (multivariate linear regression analysis) consisted of only statistically significant values (*p* < 0.05). We used the regression analysis when all independent variables are binary categorical, and the dependent variable is continuous (is normally distributed). Assumptions for linear regression were checked and met. Binary variables need no linearity assumptions, as they are already linear. To check for multicollinearity, we added collinearity diagnostics, and in order to assess autocorrelation, we added the Durbin–Watson test. The scatter plot was carried out to check whether homoscedasticity is given. R-squared (*R*^2^), F test (*F*), and overall significance of the regression model were reported (*p*). Data were analyzed using IBM SPSS 24 statistics software (IBM Corporation, New York, NY, USA).

## 3. Results

We invited 214 parents of children treated or consulted at the selected hospitals throughout the course of the research project to participate. Four parents (two mothers and two fathers) refused to participate in the study; therefore, the questionnaires were distributed to 210 parents. We received 204 correctly filled surveys, out of which 9 were excluded from the research database due to incorrect completion; hence, the overall response rate was 91%. The questionnaires with omitted individual questions, for example, unmarked number of children in the family, and the age of the child with cancer were included in the analysis, so the respondent number (*n*) per factor category might vary. We further analyzed data from 195 questionnaires.

### 3.1. Experimental-Setup Characteristics

Study participants were 195 parents, 151 mothers (77.4%), and 44 (22.6%) fathers. The mean age of the parents was 37.76 years (SD = 7.13). The age of the respondents’ children diagnosed with cancer ranged from 1 to 17 years, with a mean of 8.48 years (SD = 4.75). Of the children, 98 (50.3%) were male and 97 (49.7%) were female. The distribution of participant data by other sociodemographic and medical factors is presented in Table 1.

### 3.2. PTSS Expression among Parents of Children with Cancer

**Figure 1:**
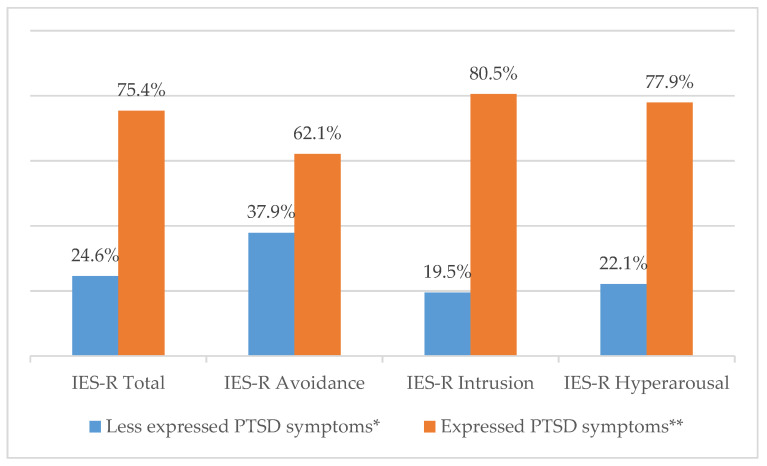
Frequency of post-traumatic stress symptoms (PTSS) among parents of children with cancer. * IES-R mean <1.5; ** IES-R mean ≥1.5.

The mean value of the total IES-R scale was 2.01 points (SD = 0.76; Table 2). The IES-R Intrusion subscale had the highest mean score (2.24; SD = 0.88), and IES-R Avoidance the lowest (1.70; SD = 0.77). From the analyzed respondent data, 147 (75.4%) parents of children with oncological disease expressed PTSD symptoms on the IES-R Total scale. When evaluating individual IES-R subscale scores, 121 (62.1%) parents exhibited avoidance, 157 (80.5%) intrusion, and 152 (77.9%) hyperarousal symptoms (Figure 1).

### 3.3. PTSS Expression in Parents of Children with Oncologic Disease by Sociodemographic Factor

Data analysis revealed that PTSS were more pronounced among mothers of children with cancer (M = 2.19) than among fathers (M = 1.40; *p* < 0.001). PTSD symptoms were more pronounced among older parents than younger ones (M = 2.17; *p* = 0.018), where the age groups were 40–64 and 19–39 years, respectively. Symptoms were also more common in professionally inactive (M = 2.16) than active (M = 1.91) parents (*p* = 0.029). In addition, PTSS were more prevalent among parents of 11–17-year-old teenagers (M = 2.21) than parents of under-10-year-olds (M = 1.91; *p* = 0.005). Moreover, symptoms were more frequent in parents directly involved in childcare (Md = 2.05) compared to indirectly involved parents (Md = 1.50; *p* < 0.001). Parents’ education level, place of residence, marital status, subjectively assessed family financial situation, number of children in the family, and the gender of the child with cancer were not related to the severity of PTSD symptoms (Table 3).

### 3.4. Parental PTSS Expression by Medical Factors of Child’s Cancer

We found a stronger link of PTSD symptom severity in parents whose child relapsed (M = 2.39) compared to participants whose children did not (M = 1.85; *p* < 0.001) (Table 4). PTSS expression differed among parents depending on the stage of child’s oncologic disease (*p* = 0.026). PTSD symptoms were the most frequent in parents caring for a child with Stage IV cancer. For example, the mean of the parents’ total IES-R scale score was MD = 2.36 when Stage IV childhood cancer was diagnosed compared to MD = 1.68 in parents whose children had Stage III cancer (*p* = 0.020). No statistically significant differences were found when comparing cancer type, disease duration, treatment method, and disease prognosis (Table 4).

### 3.5. Sociodemographic and Medical Factors Predicting Parental PTSD Symptom Expression

We used simple linear regression to determine factors that predict PTSS. PTSD symptom occurrence was correlated with parent gender (*p* < 0.001), age (*p* = 0.018), employment (*p* = 0.029), child’s age (*p* = 0.008), and relapse (*p* < 0.001). Parents’ education, place of residence, financial situation, number of children in the family, gender of the child with cancer, disease duration, and subjectively perceived prognosis of the disease were not statistically significant in this model (*p* > 0.05) (Table 5).

We included statistically significant factors associated with PTSS to the multiple linear regression model for further analysis. Obtained data showed that parent age, employment, and the age of the child with oncologic disease were no longer statistically significant in the prediction of PTSD symptoms (*p* > 0.05). Aiming to find an optimal model that broadly reflected the factors predicting PTSS expression, we integrated factors obtained from simple linear regression with lower statistical significance (*p* ≤ 0.2) into the multiple linear regression model. Tests to see if the data met the assumption of collinearity indicated that multicollinearity was not a concern (the maximum value of VIF = 1.01, which did not exceed 4, and the minimum value of tolerance = 0.942). The data met the assumption of independent errors (Durbin–Watson value = 1.96). The coefficient of determination value (*R*^2^ = 0.321, *F* = 26.84, *p* < 0.001) confirmed the suitability of the model. Consequently, three factors, namely parental gender, education, and relapse, remained statistically significant. Mothers tended to be more susceptible to post-traumatic stress than fathers (*β* = 0.83, *p* < 0.001), suggesting that the female gender may increase PTSD risk. Regardless of gender, high parental education (*β* = −0.21, *p* = 0.034) and the absence of child cancer relapse (*β* = −0.46, *p* < 0.001) seemed to reduce PTSS expression, and may play the role of protective factors.

## 4. Discussion

The diagnosis of a child’s oncologic disease is often unexpected. It may cause severe anxiety and life changes, thus becoming a major challenge for the whole family. Studies on parents’ mental health state show that when a child is diagnosed with cancer, everyday life is shaken up, and the feeling of safety is taken over by fear, uncertainty, chaos, and loneliness [27]. Learning about a child’s life-threatening disease is an event that can lead to PTSD [5,8]. However, results from previous studies on PTSS expression among parents raising children with cancer are still discordant. This study aimed to evaluate PTSS expression among Lithuanian parents caring for minors with cancer, and to assess the significance of sociodemographic and medical factors for the risk of developing PTSD.

Addressing the first goal, we found that 75.4% parents in our study group expressed PTSS. Our data supported the hypothesis that the diagnosis of a child’s cancer causes severe stress to parents, which may be accompanied by characteristic PTSD symptoms. The baseline criterion for increased risk of post-traumatic stress was a mean score of ≥1.5 on the IES-R scale. The prevalence of PTSD symptoms among parents of children with an oncologic disease can vary. A recent meta-analysis [28] indicated that PTSD occurred in 4–75% of parents whose children had cancer. The authors explained this remarkable heterogeneity by methodological differences of the analyzed studies. They varied in the choice of assessment tools and criteria with blurry application margins along with differences in elapsed time since cancer diagnosis. Some differences in the interpretation of the results could also be observed in studies that used the IES-R methodology to assess PTSS expression. The bias may have arisen due to the differential usage of mean score values on the total IES-R scale for PTSD risk evaluation: mean of ≥1.4 points or 30 points overall, mean of ≥1.5 points or 33 points overall [25,29,30], and mean of ≥1.54 points or 34 points overall [31]. This variation burdens the comparison of data among different countries. In addition, even if the same methodology is used, there is a considerable amount of confusion in the interpretation of the obtained results because of varying terminology. Some authors used the term “PTSD diagnosis”, while others described subclinical symptoms of post-traumatic stress that are inconsistent with PTSD diagnosis. These discrepancies may arise because PTSS might be more common than a clinical diagnosis of PTSD [32].

Data on the expression of PTSS and its risk factors in parents caring for children with oncologic disease are ambiguous and sometimes contradictory in the scientific literature. It is not fully determined if the age of parents raising children with oncologic disease has a significant effect on the expression of PTSD symptoms. Some findings do not confirm the association between age and PTSS [22,33], while others suggest that younger parents have more expressed symptoms [19]. Contradicting these studies, our data demonstrated a stronger PTSD symptom expression in older parents. One possible explanation for these differences is that older parents may have had more traumatic experiences and events throughout life. It is known that the risk of PTSD increases with a higher incidence of traumatic events [34,35].

It should be noted that there is still no unequivocal answer in the scientific literature about how the age of a child with cancer is related to the traumatic effect on parents. Some studies found no association between the sick child’s age and the expression of parental PTSD symptoms [33], whilst others showed a stronger expression of PTSS when the child with cancer was younger [36]. Our results once again contradict the previous findings. We found that parents raising 11–17-year-old children experienced more pronounced symptoms of PTSD than parents raising younger children. There is no straightforward explanation for the variation in these results. The relationship between age and the PTSS expression remains an open question and requires further analysis.

The childhood cancer stage is another contradictory result that encourages one to further study the psychosocial risk factors of parents raising children with oncologic disease. Data from our study showed the strongest expression of PTSS in parents whose children were diagnosed with the fourth stage of cancer. Naturally, the parental stress may increase as the child’s disease worsens. However, large differences between the PTSD symptom expression in parents of children with stage III cancer, whose symptoms were pronounced the least, and parents of children with last cancer stage are unexpected and require further investigation.

The second goal of this study was to identify which sociodemographic and medical factors are relevant in predicting PTSS expression. To begin with, we found that the female gender increased the risk of developing PTSD, while higher parental education and the absence of the child’s cancer relapse reduced the severity of PTSS. Some studies indicated that women are more likely to experience PTSD symptoms than men [37]. Throughout life, 10–12% of women and around 5% of men are diagnosed with PTSD [38,39]. As proposed by Duncan et al. (2018), genetic predisposition could influence the risk of PTSD. The authors found that, compared to men, around one-third (29%) of the studied women were at higher risk for PTSD due to genetic factors. However, we cannot play down the importance of the sociocultural aspect when interviewing both men and women about the PTSD symptoms. In many societies, masculinity is associated with strength, stoicism, and emotional control as opposed to femininity, which is defined by vulnerability and emotional expression [40]. Women are more likely to talk about discomfort, symptoms, and difficulties than men are. For this reason, women may be diagnosed with PTSD more often than men, who often hide possible symptoms from others. Nevertheless, further research is necessary to investigate these assumptions. The primary caretaker role for a child with cancer could also explain why women are more susceptible to developing PTSS [9,41]. In our study group, mothers were principal caretakers (87.3%), while fathers tended to be periodically involved. It was observed that parents actively involved in childcare have to constantly make important treatment-related decisions; therefore, they are more likely to feel anxious about the child’s relapse, treatment methods, and endured pain [10,42,43]. Furthermore, parents spend a lot of time in hospitals while caring for a sick child, so they might be exposed to diverse traumatic experiences, including the death of other children. The accumulated fatigue caused by childcare can also contribute to the prevalence and manifestation of PTSS.

Moreover, we found that the absence of the child’s cancer relapse reduced the likelihood of developing PTSD symptoms. We assume that the parents might perceive the absence of relapse as a successful management of the disease. Relapse is commonly associated with an increased risk of the child’s death and treatment ineffectiveness. According to Dunn et al. (2012) [14], symptoms of post-traumatic stress were more pronounced in parents whose children experienced relapse than in parents whose children were treated for the first time. In addition, a study by Jurbergs et al. (2009) [17] demonstrated that relapse is a significant prognostic factor in parental PTSD.

In addition to these results, we found that higher parental education reduced the severity and likelihood of PTSS. Research on PTSD symptom expression with respect to parental education is still conflicting. Some studies claim that highly educated parents are more likely to experience severe PTSS than less educated ones [19]. However, others associate a lower education level with more pronounced symptoms of post-traumatic stress [14]. It is difficult to interpret these findings; hence, further research is necessary to elucidate the relevant factors of parental education that play a role in coping with childhood cancer-related stress. Our data suggested that parental education is associated with a lower risk of developing PTSS. We hypothesize that education is one of the protective factors against PTSD risk. The protective effect against traumatic stress can be based on the assumption that parents with higher education easily understand medical information and may have better access to the newest science-based information on cancer risks and treatments. Highly educated parents often have bigger salaries, granting the possibility to consult other health professionals, and access a wider range of options of child treatment and rehabilitation. For these reasons, parents with higher education may feel in tighter control of the situation when facing their child’s cancer. All of the above-mentioned factors may be protective against the traumatic effects associated with the child’s disease.

We would like to address a couple of limitations of this research study. First, possible traumatic events in the parents’ lives other than the child’s cancer were not included and may have exacerbated the severity of PTSS. A differently designed research project including the longer observation of study participants would allow for a better explanation of specific factor contributions in increased or decreased risk of PTSD. Second, data regarding the child’s disease and treatment were obtained directly from the parents, and medical records were not consulted; hence, some bias might have been introduced. All in all, these are minor limitations that did not affect the overall quality of the study.

## 5. Conclusions

In summary, the study is a relevant advancement in the field of psycho-oncology. Although the link between the mental health of parents caring for children with cancer and the risk of PTSD is widely globally studied, it is the first study addressing this problem in Lithuania. Our results demonstrated that a child’s oncologic disease is an extremely stressful event for many parents that may impair their mental health and increase the risk of developing PTSD. Our data are particularly useful in outlining psycho-oncologic care and organizing the necessary support for parents raising minors with cancer.

## Figures and Tables

**Table 1:** Respondent data by sociodemographic and medical factors (*n* = 195).

Factors		*n* (%)
Gender	Male	44 (22.6)
	Female	151 (77.4)
Age (years)	19–39	121 (62.1)
	40–64	74 (37.9)
Education	Higher education	112 (57.4)
	Below higher education	83 (42.6)
Place of residence	Urban	128 (65.6)
	Rural	67 (34.4)
Employment	Professionally active	118 (60.5)
	Professionally inactive	77 (39.5)
Financial situation	Good	44 (22.6)
	Worse than good	150 (76.9)
Marital status	In couple	168 (86.2)
	Single	27 (13.8)
Number of children in the family	1	58 (29.7)
	2	88 (45.1)
	3 and more	48 (24.6)
Involvement in childcare	Direct (permanent)	157 (80.5)
	Indirect (periodic)	38 (19.5)
Gender of child with cancer	Male	98 (50.3)
	Female	97 (49.7)
Age of child with cancer (years)	1–10	128 (65.5)
	11–17	65 (33.3)
Cancer type	Blood and lymph	82 (42.1)
	Central nervous system	47 (24.1)
	Solid tumor	66 (33.8)
Disease duration	Shorter than one year	106 (54.4)
	Longer than one year	89 (45.6)
Cancer stage	I	9 (4.6)
	II	29 (14.9)
	III	29 (14.9)
	IV	31 (15.9)
	Not specified	78 (40.0)
Treatment method	Surgery	5 (2.6)
	Chemotherapy	79 (40.5)
	Radiotherapy	12 (6.2)
	Bone-marrow transplantation	8 (4.1)
	Follow-up after treatment	87 (44.6)
	Chemotherapy and radiotherapy together	4 (2.1)
Relapse	Occurred	46 (23.6)
	Did not occur	128 (65.6)
Subjectively perceived treatment prognosis	Good	55 (28.2)
	Bad	111 (56.9)

**Table 2:** Impact of Event Scale-Revised (IES-R) and individual subscale scores.

IES-R	*N*	Minimum	Maximum	Mean	Standard Deviation (SD)
IES-R Total	195	0	3.86	2.01	0.76
IES-R Avoidance	195	0	3.75	1.70	0.77
IES-R Intrusion	195	0	4.00	2.24	0.88
IES-R Hyperarousal	195	0	4.00	2.13	0.96

**Table 3:** Comparison of Impact of Event Scale-Revised (IES-R) results by sociodemographic factor.

Sociodemographic Factor		IES-R Total		*p*
		Mean (SD)		
Gender	Male	2.19 (0.68)		<0.001
	Female	1.40 (0.72)		
Age (years)	19–39	1.91 (0.74)		0.018
	40–64	2.17 (0.77)		
Education	Higher education	1.94 (0.81)		0.127
	Below higher education	2.10 (0.69)		
Place of residence	Urban	2.02 (0.73)		0.842
	Rural	1.99 (0.81)		
Employment	Professionally active	1.91 (0.78)		0.029
	Professionally inactive	2.16 (0.70)		
Financial situation	Good	1.91 (0.72)		0.337
	Worse than good	2.04 (0.77)		
Number of children in the family	1–2	1.96 (0.73)		0.172
	3 and more	2.13 (0.84)		
Gender of child with cancer	Male	1.96 (0.72)		0.339
	Female	2.06 (0.79)		
Age of child with cancer (years)	1–10	1.91 (0.80)		0.005
	11–17	2.21 (0.65)		
		**Median** **(min; max)**	**Mean Rank**	***p***
Marital status	In couple	2.00 (0.00; 3.86)	95.79	0.173
	Single	2.00 (1.36; 3.48)	111.74	
Involvement in childcare	Direct (permanent)	2.05 (0.05; 3.86)	106.25	<0.001
	Indirect (periodic)	1.50 (0.00; 3.45)	63.93	

**Table 4:** Comparison of Impact of Event Scale-Revised (IES-R) results by medical factors of child’s cancer.

**Medical Factors**		**Mean (SD) of Total IES-R**		***p***
Cancer type	Blood and lymph	2.00 (0.76)		0.988
	Central nervous system	2.00 (0.78)		
	Solid tumor in different organs	2.02 (0.75)		
Disease duration	Shorter than one year	1.97 (0.76)		0.495
	Longer than one year	2.05 (0.76)		
Relapse	Occurred	2.39 (0.59)		<0.001
	Did not occur	1.85 (0.78)		
Subjectively perceived treatment prognosis	Good	1.87 (0.77)		0.106
	Bad	2.08 (0.79)		
**Medical Factors**		**Median (min; max)**	**Mean Rank**	***p***
Treatment method	Surgery	2.27 (1.73; 3.27)	127.4	0.162
	Chemotherapy	1.90 (0.00; 3.36)	87.31	
	Radiotherapy	1.90 (1.36; 2.36)	79.46	
	Bone-marrow transplantation	2.18 (1.50; 2.68)	109.06	
	Follow-up after treatment	2.05 (0.68; 3.86)	103.17	
Cancer stage	I	2.00 (0.68; 2.91)	85.78	0.026
	II	2.14 (1.23; 3.36)	104.55	
	III	1.68 (1.05; 3.48)	76.78	
	IV	2.36 (0.05; 3.27)	107.37	
	Not specified	1.98 (0.00; 3.86)	79.71	

**Table 5:** Factors predicting parental PTSS by simple and multiple linear regression models.

Factors	Simple Linear Regression			Multiple Linear Regression			
	*β*	95% CI	*p*	*β*	95% CI	*β*_s_	*p*
Gender (female vs. male)	0.79	0.56; 1.00	<0.001	0.83	0.60; 1.00	0.45	<0.001
Age (years; 40−64 vs. 19−39)	0.27	0.05; 0.49	0.018	-	-	-	-
Education (higher education vs. below higher education)	−0.17	−0.38; 0.52	0.135	−0.21	−0.41; −0.17	−0.14	0.034
Place of residence (rural vs. urban)	−0.02	−0.25; 0.21	0.842	-	-	-	-
Employment (professionally active vs. professionally inactive)	0.24	0.03; 0.46	0.029	-	-	-	-
Financial situation (worse than good vs. good)	0.13	−0.13; 0.39	0.337	-	-	-	-
Number of children in the family (3 or more vs. 1−2)	0.17	−0.08; 0.43	0.172	-	-	-	-
Gender of child with cancer (female vs. male)	0.11	−0.11; 0.32	0.339	-	-	-	-
Age of child with cancer (years; 11−17 vs. 1−10)	0.31	0.08; 0.53	0.008	-	-	-	-
Disease duration (longer vs. shorter than one year)	0.08	−0.14; 0.29	0.495	-	-	-	-
Relapse (did not occur vs. occurred)	−0.54	−0.78; 0.29	<0.001	−0.48	−0.69; −0.26	−0.27	<0.001
Subjectively perceived treatment prognosis (good vs. bad)	−0.21	−0.47; 0.05	0.106	-	-	-	-
